# *Atractylodes chinensis* volatile oil up-regulated IGF-1 to improve diabetic gastroparesis in rats 

**DOI:** 10.22038/IJBMS.2022.60126.13339

**Published:** 2022-04

**Authors:** Hongzeng Li, Yitong Wang, Yuxin Tian, Feiyue Tian, Zhiyang Xing, Yunfei Wang, Meixing Yan, Yanling Gong

**Affiliations:** 1 Department of Pharmacy, College of Chemical Engineering, Qingdao University of Science and Technology, Qingdao, China; 2 Shandong Xinhua Pharmaceutical Company Limited, Zibo, China; 3 Qingdao Women and Children’s Hospital, Qingdao, China; # These authors contributed equally to this work

**Keywords:** Atractylodes, Gastroparesis, Insulin-like growth factor I, Interstitial cells of cajal, Stem cell factor

## Abstract

**Objective(s):**

Diabetic gastroparesis (DGP) is one of the main complications of diabetes, and more than half of diabetes cases are accompanied by gastroparesis. This study aims to explore the effect of *Atractylodes chinensis *volatile oil (ACVO) on DGP rats.

**Materials and Methods:**

The rats were injected with STZ combined with a high-sugar and high-fat diet in an irregular manner to establish the DGP model. ACVO at different doses (9.11 mg/kg, 18.23 mg/kg, and 36.45 mg/kg) were given by intragastric administration. A mixture of cisapride and metformin was used as the positive control. At the end of the experiment, gastric emptying and intestinal propulsion were determined. Then the tissue samples and blood were taken from each group for serum analysis, western blot and immunopathological examination.

**Results:**

After treatment with ACVO, body weight increased and blood glucose decreased when compared with rats in the DGP group. Gastric emptying and intestinal propulsion were accelerated, and gastric acid secretion increased. The serum insulin-like growth factor-1 (IGF-1) level was increased. Protein expressions and positive cells of IGF-1 receptor (IGF-1R), acetylcholine transferase (CHAT), and stem cell factors (SCF) in the stomach were significantly increased determined by western blot and immunofluorescence staining. The morphology and the number of interstitial cells of Cajal (ICCs) in the stomach were restored, determined by hematoxylin and eosin staining and immunohistochemical staining, respectively.

**Conclusion:**

ACVO effectively alleviated DGP in rats, and its mechanism may be related to the up-regulation of IGF-1/IGF-1R signaling.

## Introduction

Diabetic gastroparesis (DGP) is a complication of diabetes and about 50% of people with diabetes suffer from it. Some DGP patients have no obvious symptoms, and some patients will have accompanying indigestion and delayed gastric emptying (1), characterized by early satiety and persistent nausea. DGP not only brings physical pain to the patients but also reduces the quality of life and happiness of the patients. Drug therapy and dietary changes are helpful to increase gastric motility and improve the symptoms of gastroparesis. However, adverse reactions caused by drugs have greatly limited the development and use of gastric motility drugs (2-4). Therefore, it is particularly important to develop a new drug with low toxicity and side effects for the treatment of DGP.

The volatile oil of *Atractylodes chinensis* (ACVO) is extracted from the rhizome of *A. chinensis*. Studies have found that ACVO has high medicinal values such as antibacterial, anti-hypoxia, hypoglycemic, anti-ulcer, diuretic, anti-gastritis, etc (5-8). Sesquiterpenes, the main components of ACVO, have obvious anti-inflammatory and insecticidal activity (9, 10). Therefore, ACVO is widely used in the fields of medicine and agriculture. Some studies have shown that *A. chinensis* rhizome can be used to treat gastrointestinal diseases (11). Its volatile oil composition has a potential effect for the treatment of colitis (colitis syndrome) (12). However, the effects of volatile oil from *A. chinensis* on DGP and its mechanism have not been reported.

Studies have confirmed that the absence of interstitial cells of Cajal (ICCs) eventually leads to DGP (13). ICCs are a pacemaker in the gastrointestinal tract that triggers gastric motility. Meanwhile, ICCs are an intermediate that conduct neurotransmission from the enteric nervous system to the gastric smooth muscle. Stem cell factor (SCF) is responsible for phenotypic maintenance, proliferation, and differentiation of ICCs (14). Insulin-like growth factor-1(IGF-1) is a homologous molecule that promotes cell growth and differentiation and is also a nutrient for neurons (15). Excitatory neuron acetylcholine transferase (CHAT) and inhibitory neuron nitric oxide synthase (nNOS) work together to balance gastric motility. In previous studies, it was found that nitric oxide synthase is abnormal in DGP. However, there is less research on CHAT. It has been reported that IGF-1 has a significant improvement on peripheral neuropathy caused by diabetes (16, 17). The damage to its receptor (IGF-1R) promotes the development of DGP (18).

In this study, rats were injected with streptozocin and fed with high sugar and fat to establish a DGP model, and the effect of ACVO on DGP involved in IGF-1 signaling was explored. The results might lay a foundation for the development of new drugs to treat DGP.

## Materials and Methods

***Materials and reagents***

ACVO was extracted from the dried rhizome of *A. chinensis*, which was purchased from Qingdao Guofeng Pharmacy (Qingdao, Shandong, China). Cisapride and metformin were purchased from Shandong Qikang Pharmaceutical Co., Ltd. (Dezhou, Shanodong, China). Streptozotocin (STZ), enzyme-linked immunosorbent assay (ELISA) kits for serum IGF-1, and Bicinchoninic acid (BCA) kits were obtained from Beijing Solarbio Science & Technology Co., Ltd. (Beijing, China). Phenolic red solutions were purchased from the Tianjin Institute of Light and Fine Chemical Engineering (Tianjin, China). IGF-1R, CHAT, and SCF antibodies were purchased from Abcam (Cambridge, UK). Sodium dodecyl sulfate polyacrylamide gel, polyvinylidene difluoride (PVDF) membranes, and ECL substrate kit were purchased from Beijing Bioss Biotechnology Co. Ltd. (Beijing, China). All other chemicals used were of analytical grade.

***Experimental animals***

One hundred and eighty-five adult male Wistar rats (180–200 g) were used in this study which were purchased from Qingdao Daren Fortune Animal Science And Technology Co., Ltd. (Qingdao, Shandong, China). The animals were placed in cages with a temperature of 22 ± 2 °C and humidity of 50–60% and fed a standard diet for 7 days of adaption. All animal experiments were approved by the Animal Care and Use Committee at the Qingdao University of Science and Technology (approval code: 20201015). The research involving animals was conducted according to the Guide for the Care and Use of Laboratory Animals.

***Animal processing***

At the end of adaptive feeding, the rats were randomly divided into the control group (NCG) (n=35) and the diabetic gastroparesis group (DGP) (n=155). Rats with DGP were intraperitoneally injected with STZ at a dose of 50 mg/kg, and rats with NCG were given the same volume of normal saline. Fasting blood glucose was determined 72 hr later. Fasting blood glucose of rats higher than 16.7 mmol/l for two consecutive weeks indicated that the rat model of diabetes was successfully established. Otherwise, STZ was supplemented.

After establishment of diabetes, the rats in the DGP group were fed a high-sugar and high-fat diet (regular diet: lard stearin : sucrose : milk powder : egg = 58 : 15 : 20 : 5 : 2) in an irregular manner (morning in odd days and afternoon in even days) while the NCG rats were fed a normal diet. Eight weeks later, five rats were randomly selected from each group to compare their appearance, diet, feces, and gastric emptying to determine the establishment of DGP. 

The successfully established DGP rats were randomly divided into 5 groups (n=30): DGP, ACVO low dose group (ACVO.L), ACVO medium-dose group (ACVO.M), ACVO high dose group (ACVO.H), and a mixture of cisapil and metformin administration group (CMG). The rats in ACVO.L, ACVO.M, and ACVO.H groups were given ACVO daily (9.11 mg/kg, 18.23 mg/kg, and 36.45 mg/kg, respectively) by intragastric administration via a specially designed gavage needle once a day for 4 weeks. The rats in the CMG group were given a mixture of cisapride (3.5 mg/kg) and metformin (175 mg/kg) while those in the NCG and DGP groups were given the same dose of saline. The weight changes of rats in each group were recorded before and after treatment.

***Rate of gastric emptying and intestinal propulsion***

After an overnight fast, 15 rats in each group were orally administered 2 ml phenolic red solution (0.05 mg/l) which was used as a marker for measuring gastric emptying and intestinal propulsion (19, 20). Twenty minutes later, the rats were anesthetized to death with pentobarbital sodium (65 mg/kg). The stomach was removed and cut open along the greater curvature. The contents were poured into a beaker and 20 ml NaOH solution (0.5 mol/l) and 0.5 ml 20% trichloroacetic acid were added. Then the supernatant was obtained by centrifugation and its absorbance was determined at 560 nm with a Microplate reader (Infinite m plex, Tecan, Switzerland). The same method was used to determine the absorbance of the standard phenolic red solution. The gastric emptying rate was calculated according to the following equation(1)(20). At the same time, the whole intestine was straightened, cut open, and NaOH was added (0.5 mol/l). Subsequently, the phenol red advancing part turned purple. The lengths of the whole intestine and the purple part were measured, and the intestinal propulsion rate was calculated according to equation (2)(20).

$\mathrm{gastric emptying rate}\left(\%\right)=(1-A_{0}/A_{1})\times 100$


(1)

A_0_ represents the absorbance of the phenolic red solution in the stomach of each group, and A_1_ represents the absorbance of standard phenolic red solution

$\mathrm{Intestinal prolulsion rate}\left(\%\right)=\frac{\mathrm{length of the purple part ofl intestine}}{\mathrm{lenght of the whole intestione}}\times 100\%$

(2)

***Fasting blood glucose, serum IGF-1, gastric juice volume, and gastric acid***

The remaining rats were used for determination of fasting blood glucose, serum IGF-1, and gastric acid. After measuring the fasting blood glucose, the rats were anesthetized with pentobarbital sodium (65 mg/kg), and then the blood was collected from the heart to separate serum by centrifugation. Serum IGF-1 concentration was determined by the ELISA method. Thereafter, the entire stomach was taken out and the blood was wiped off. Cut along with one side of the great bend of the stomach to collect the gastric juice and centrifuged at 3500 rpm/min. The supernatant was the gastric juice volume. 2 ml supernatant gastric juice was taken out and added with two drops of phenolphthalein reagent, and then titrated with 0.02 mol/l NaOH in a burette until the solution turned red with no more color change. Then the volume of the consumed NaOH solution was recorded. The gastric acid excretion amount of the rats was calculated according to the following equation(3)(21).

gastric acid secretion μEqL=VNaOH×∅

 (3)

***Western blotting***

The same part of fresh stomach tissues of rats in each group was taken and protein lysates were added. After 30 min, the homogenate was centrifuged at 12000 rpm at 4 ℃ for 10 min. The BCA kit was used to determine the protein concentration. The protein samples were separated by sodium dodecyl sulfate polyacrylamide gel (SDS-PAGE) and transferred to the PVDF membrane. Then, the protein samples were sealed with 5% skimmed milk powder for 2 hr and washed with TBST. Subsequently, the primary antibody against IGF-1R (rabbit polyclonal, 1:1000 dilution), CHAT (rabbit polyclonal, 1:1000 dilution), SCF (rabbit polyclonal, 1:1000 dilution) was added and incubated for 2 hr. Then the membranes were washed with TBST 5 times and 5 min each time. HRP-conjugated secondary antibody (1:1000 dilution) was added and incubated for 1 hr. Finally, ECL was applied evenly to the membranes for development. The membranes were imaged using an automated imaging system (Bio-Rad Gel Doc EZ, Bole Life Medicine Products Co. Ltd, Shanghai, China). Image J was used to analyze the gray value of the bands with GAPDH as the internal reference.

***Immunohistochemical staining***

The same part of the stomach was soaked in 4% paraformaldehyde, embedded in paraffin, sectioned, and then antigen repaired after dewaxing. The treated slices were incubated with 3% H_2_O_2_ for 20 min and washed with PBS three times, 5 min each time. The tissues were then sealed with goat serum for 10 min, rabbit anti-mouse C-Kit antibody (1:100) was added, and then incubated at 4 °C overnight. After washing with PBS, goat anti-rabbit secondary antibody (1:1000) labeled with biotin was added to the tissue and incubated at room temperature for 20 min. Then 3, 3-diaminobenzidine (DAB) was added and incubated for 8 min, and the slices were dyed with hematoxylin. After being sealed with neutral gum, the slices were observed under a microscope (BX53, Olympus, Tokyo, Japan).

***Hematoxylin and eosin staining***

Slices of rat stomach tissue were taken and stained with hematoxylin and eosin (H&E) according to standard operating procedure (22).

***Immunofluorescence staining***

Gastric tissue was removed after perfusion fixation and soaked in 4% paraformaldehyde for 6–8 hr, and then transferred to 30% sucrose solution. The same part of gastric tissue was frozen and then sectioned. The prepared slices were sealed with goat serum for 2 hr, then primary antibody against IGF-1R (rabbit polyclonal, 1:200 dilution), CHAT (rabbit polyclonal, 1:200 dilution), SCF (rabbit polyclonal, 1:200 dilution) was added and incubated at 4 °C overnight. The sections were cleaned with PBS 3 times, 5 min each time, and then incubated with secondary antibody (cy3-conjugated or FITC-conjugated, 1:100) for 2 hr. Finally, the sections were sealed and observed under a microscope (BX53, Olympus, Tokyo, Japan).

***Statistical analysis***

The results were presented in the form of mean ± standard deviation, and then one-way ANOVA was performed using SPSS. LDS and S-N-K were used for pairwise comparisons. When *P*≤0.05, it was considered statistically significant.

## Results

***Effects of ACVO on body weight and blood glucose in DGP rats***

**Figure 1:**
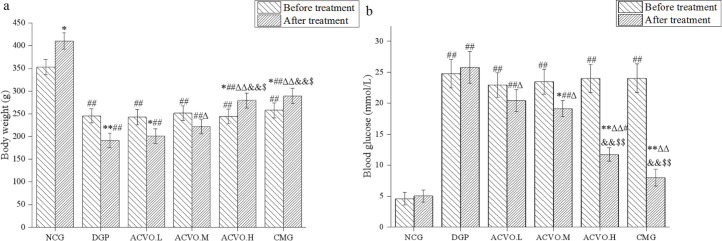
Effects of *Atractylodes chinensis *volatile oil (ACVO) on body weight and blood glucose in diabetic gastroparesis (DGP) rats. n = 15; *P<0.05, **P<0.01, compared with before the treatment; #*P*<0.05, ##*P*<0.01, compared with the control group; diabetic gastroparesis *P*<0.05, ΔΔ*P*<0.01, compared with the diabetic gastroparesis (DGP) group; &*P*<0.05, &&*P*<0.01, compared with the *Atractylodes chinensis *volatile oil low dose (ACVO.L) group; $*P*<0.05, $$*P*<0.01, compared with the *Atractylodes chinensis *volatile oil medium-dose (ACVO.M) group

At the end of the experiment, there was a significant decrease in body weight and an increase in blood glucose in the DGP group compared with the NCG group (Figure 1a and b, *P*<0.01), indicating successful establishment of diabetes. During the whole experiment, NCG rats fed a normal diet showed a steady weight gain (Figures 1a and b, compared with before treatment, *P*<0.01) and no significant changes in blood glucose (Figures 1a and b, compared with before treatment, *P*>0.05). However, the weight of DGP rats decreased significantly and blood glucose increased significantly (Figures 1a and b, compared with before treatment, *P*<0.01). After the ACVO treatment, the weight of rats in ACVO.L, ACVO.M, and ACVO.H groups increased significantly while the blood glucose decreased significantly (Figures 1a and b, compared with before treatment in the same group, *P*<0.05 or *P*<0.01). Furthermore, with the increase in dose, the effect of ACVO increased (Figure 1a and b, compared with the DGP group after treatment, *P*<0.05 or *P*<0.01) and the effect of a high dose of ACVO was similar to that of CMG. The results showed that ACVO exhibited a certain hypoglycemic effect.

***Effects of ACVO on gastric emptying, intestinal propulsion, gastric acid, and gastric juice volume in DGP rats***

**Figure 2:**
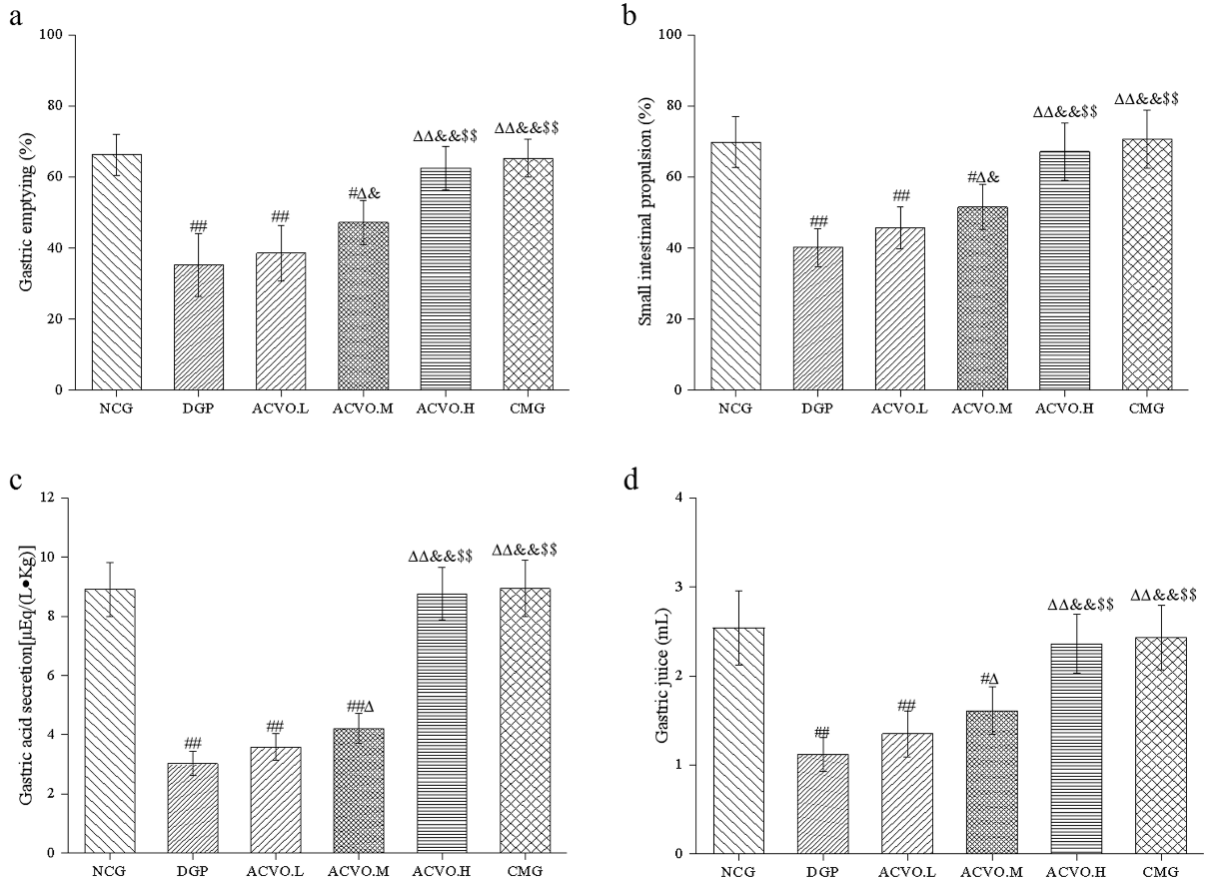
Effects of *Atractylodes chinensis* volatile oil (ACVO) on gastric emptying, intestinal propulsion, and gastric acid in diabetic gastroparesis (DGP) rats. n=15; *P<0.05, **P<0.01, compared with before the treatment; #*P*<0.05, ##*P*<0.01, compared with the control group; Δ*P*<0.05, ΔΔ*P*<0.01, compared with the diabetic gastroparesis (DGP) group; &*P*<0.05, &&*P*<0.01, compared with the *Atractylodes chinensis* volatile oil low dose (ACVO.L) group;; $*P*<0.05, $$*P*<0.01, compared with the *Atractylodes chinensis* volatile oil medium-dose (ACVO.M) group

Compared with the NCG group, DGP rats had significantly slower gastric emptying, intestinal propulsion, less gastric acid secretion, and gastric juice volume, (Figures 2a, b, c, and d, *P*<0.01), which was similar to the clinical features of DGP. However, gastric emptying rate, intestinal propulsion rate, gastric acid secretion, and gastric juice volume increased in the rats treated with ACVO, showing a dose-dependent manner (Figures 2a, b, c, and d, compared with the DGP group, *P*<0.05 and *P*<0.01). The high dose of ACVO exhibited a similar effect with the CMG, showing no significant difference with the NCG group (Figures 2a, b, c, and d).

***Effect of ACVO on serum IGF-1 level in DGP rats***

**Figure 3:**
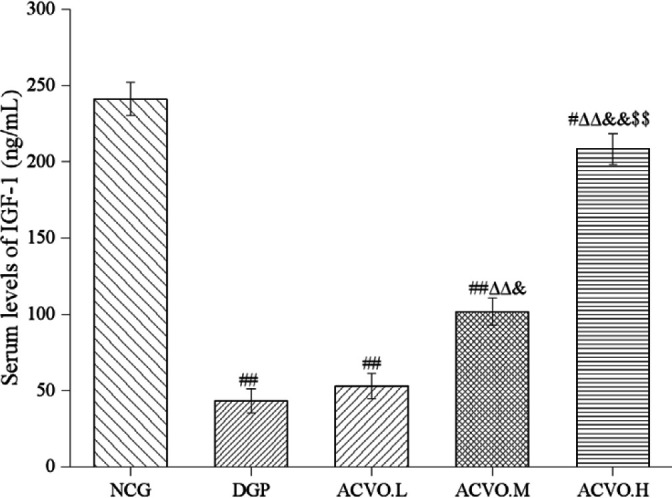
Effect of *Atractylodes chinensis* volatile oil (ACVO) on serum insulinlike growth factor-1 (IGF-1) level in diabetic gastroparesis (DGP) rats. n=15; #*P*<0.05, ##*P*<0.01, compared with the control group; Δ*P*<0.05, ΔΔ *P*<0.01, compared with the diabetic gastroparesis (DGP) group; &*P*<0.05, &&*P*<0.01, compared with the Atractylodes chinensis volatile oil low dose (ACVO.L) group; $*P*<0.05, $$*P*<0.01, compared with the Atractylodes chinensis volatile oil medium-dose (ACVO.M) group

Compared with the NCG group, the serum IGF-1 level in the DGP rats was significantly reduced (Figure 3, *P*<0.01). After treatment with ACVO, the serum IGF-1 level of rats was gradually increased in a dose-dependent manner (Figure 3, compared with the DGP group, *P*<0.01).

***Effect of ACVO on IGF-1R, CHAT, and SCF protein expression in DGP rats***

**Figure 4:**
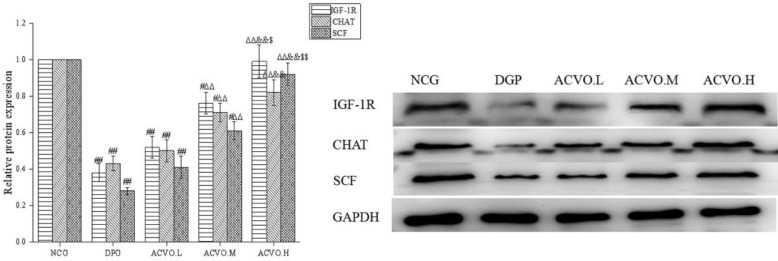
Effect of *Atractylodes chinensis *volatile oil (ACVO) on insulin-like growth factor-1 (IGF-1R) receptor, acetylcholine transferase (CHAT), and stem cell factor (SCF) protein expression in diabetic gastroparesis (DGP) rats. n=15; #*P*<0.05, ##*P*<0.01, compared with the control group; Δ*P*<0.05, ΔΔ*P*<0.01, compared with the diabetic gastroparesis (DGP) group; &*P*<0.05, &&*P*<0.01, compared with *Atractylodes chinensis *volatile oil low dose (ACVO.L) group; $*P*<0.05, $$*P*<0.01, compared with the *Atractylodes chinensis *volatile oil medium-dose (ACVO.M)group

Compared with the NCG group, the protein expressions of IGF-1R, CHAT, and SCF in the gastric tissues of DGP rats were significantly decreased (Figures 4a and 4b, *P*<0.01). After treatment with ACVO, IGF-1R, CHAT, and SCF protein expressions in the gastric tissue increased with the increase of the dose of ACVO, showing statistical significance in the medium and high dose group (Figures 4a and 4b, compared with the DGP group, *P*<0.01). 

***Effect of ACVO on the immune activity of IGF-1R, CHAT, and SCF in DGP rats***

**Figure 5:**
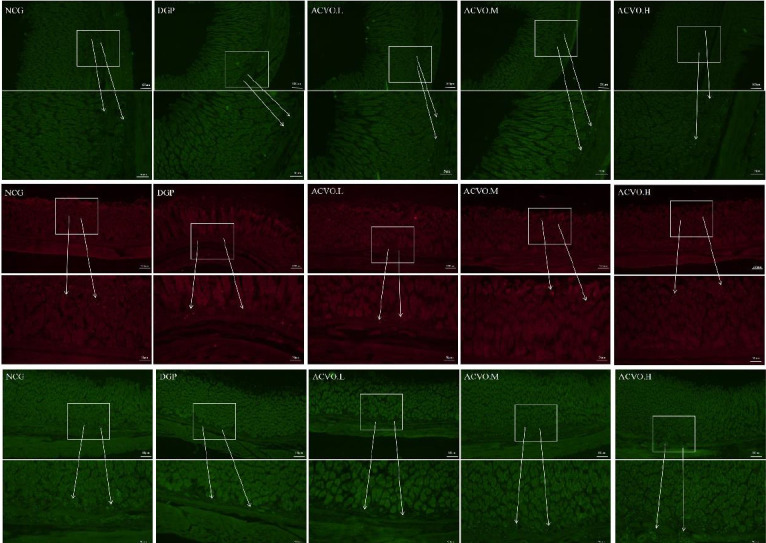
Effect of *Atractylodes chinensis* volatile oil (ACVO) on the immune activity of insulin-like growth factor-1 receptor (IGF-1R), acetylcholine transferase (CHAT), and stem cell factor (SCF) in diabetic gastroparesis (DGP) rats. a: IGF-1R; b: SCF; c: CHAT

Immunofluorescence was used to analyze the expression of IGF-1R, CHAT, and SCF in the gastric tissues of rats in each group. The results revealed that the immunopositive reaction of IGF-1R, CHAT, and SCF in the gastric tissues of DGP rats was significantly reduced when compared with the NCG group (Figures 5a, b, and c). However, the immunopositive reactions of IGF-1R, CHAT, and SCF were both increased with the increase in ACVO dose (Figures 5a, b, and c).

***Effect of ACVO on gastric histomorphology of DGP rats***

**Figure 6:**
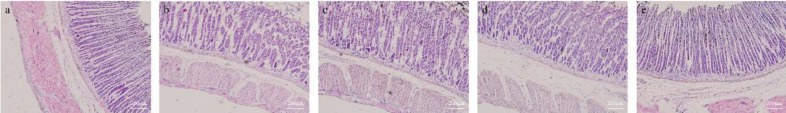
Effect of *Atractylodes chinensis* volatile oil (ACVO) on gastric histomorphology of diabetic gastroparesis (DGP) rats. a: the control group; b: the diabetic gastroparesis (DGP) group; c: Figure 6. Effect of *Atractylodes chinensis * volatile oil on gastric histomorphology of diabetic gastroparesis rats. a: the control group; b: the diabetic gastroparesis group; c: the *Atractylodes chinensis *volatile oil low dose group; d: the Atractylodes chinensis volatile oil medium-dose group; e: the *Atractylodes chinensis *volatile oil high dose group. The gastric tissues of the the control group rats were intact without any significant damage. The cells were orderly arranged, and there was no inflammatory infiltration, showing a normal morphology (Figure 6a). In contrast, the gastric tissue cells of the diabetic gastroparesis rwere disordered and some of the cells were deformed (Figure 6b). Compared with the diabetic gastroparesis group, the morphology of rat gastric tissue cells in the *Atractylodes chinensis *volatile oil treatment group was improved to varying degrees (Figures 6c, 6d, and 6e)

The results of HE staining were shown in Figure 6. The gastric tissues of the NCG rats were intact without any significant damage. The cells were orderly arranged, and there was no inflammatory infiltration, showing a normal morphology (Figure 6a). In contrast, the gastric tissue cells of DGP rats were disordered and some of the cells were deformed (Figure 6b). Compared with the DGP, the morphology of rat gastric tissue cells in ACVO treatment groups was improved to varying degrees (Figures 6c, 6d, and 6e) With the increase of the dose, the morphology of gastric tissue was gradually restored to normal. There was no significant difference between ACVO. H and NCG groups (Figures 6a and 6e).

***Effect of ACVO on c-kit expression of ICCs in the gastric tissues of DGP rats***

**Figure 7:**
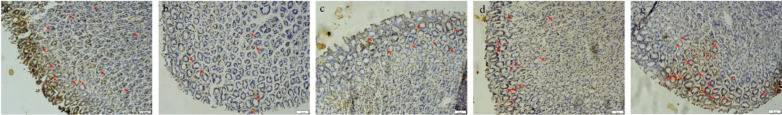
Effect of *Atractylodes chinensis* volatile oil on C-kit expression of interstitial cells of Cajal in the gastric tissue of diabetic gastroparesis rats.a: the control group; b: the diabetic gastroparesis group; c: the *Atractylodes chinensis *volatile oil low dose group; d: the *Atractylodes chinensis *volatile oil medium-dose group; and e: the *Atractylodes chinensis *volatile oil high dose group. R e d arrows: interstitial cells of Cajal positive cells. Compared with the control group, the expression of interstitial cells of Cajal-positive cells in the gastric tissues of diabetic gastroparesis rats was significantly decreased (Figures 7a and 7b). After the treatment of *Atractylodes chinensis *volatile oil, the expression of interstitial cells of Cajal positive cells in the gastric tissues of diabetic gastroparesis rats was up-regulated to varying degrees with the increase of the dose (Figures 7c, 7d, and 7e)

The expression of c-kit in the gastric tissues of the rats in each group was shown in Figure 7. Compared with the NCG group, the expression of ICCs positive cells in the gastric tissues of the DGP rats was significantly decreased (Figures 7a and 7b). After the treatment of ACVO, the expression of ICCs positive cells in the gastric tissues of DGP rats was up-regulated to varying degrees with the increase of the dose (Figures 7c, 7d, and 7e). The results suggested that ACVO could restore ICCs positive cells in the gastric tissues of DGP rats.

## Discussion

The link between gastroparesis and diabetes has been noted for the last century, and DGP is a serious complication of diabetes. However, DGP is less likely to be detected before the onset of the disease and is difficult to treat (23). According to recent reports, some Chinese herbal ingredients can effectively treat DGP (24). In this study, we explored the therapeutic effect of ACVO on DGP rats and its potential mechanism. The results showed that ACVO significantly reduced blood glucose and promoted gastrointestinal motility in DGP rats. The mechanism of its effect may be related to elevation of serum IGF-1, up-regulation of IGF-1R, CHAT, SCF expression, and the restoration of ICCs number positive cells in the gastric tissues.

The DGP model induced by intraperitoneal injection of STZ and irregular high-sugar and high-fat diet is a widely used modeling method (25). DGP is characterized by weight loss, increased blood glucose, delayed gastric emptying, and reduced excretion of gastric acid. Gastric acid is the main substance that activates pepsinogen in the gastric juice converting it to active pepsin, which digests food in the acidic environment provided by gastric acid, thereby promoting gastric emptying. In DGP rats, a sharp drop in body weight and a significant increase in blood sugar were intuitive indicators of DGP. Gastric emptying was delayed accompanied by a significant reduction in intestinal propulsion. After the treatment of ACVO, the body weight and blood glucose were recovered, and the status of delayed gastric emptying and reduced intestinal propulsion rate were also significantly alleviated. To our knowledge, the therapeutic effect of ACVO on DGP is revealed for the first time.

ICCs are a kind of mesenchymal cells which are scattered in the gastrointestinal smooth muscle and autonomic nerve endings. It is recognized as a pacemaker to transduce electrical activity to the gastric smooth muscle and trigger muscle contraction. C-kit protein is the main protein expressed in ICCs (26). A decrease in C-Kit expression indicates ICCs abnormalities, resulting in gastric emptying delay (27). 

IGF-1 acts as a nutritional factor that promotes the growth of cells in the gastrointestinal tract. IGF-1R is activated by IGF-1. Activation of the IGF-1R stimulates multiple pathways which finally results in multiple biological effects in a variety of tissues and cells. IGF-1/IGF-1R signaling was implicated in the development of the nervous system by promoting neuronal growth, survival, proliferation, and differentiation. Previous studies have shown that there is no direct relationship between ICCs and IGF-1. However, IGF-1 is expressed in gastric smooth muscle cells and induces gastric smooth muscle cells to produce SCF (27-29). SCF is a ligand of C-Kit and plays an important role in the phenotypic differentiation and maintenance of ICCs. The SCF/ C-Kit pathway is the main way to regulate ICCs (30). The decrease in IGF-1 signal transduction leads to a decrease in SCF expression, which leads to depletion of ICCs (31). In our present study, the influence of ACVO on IGF-1 and ICCs has been explored. The results showed that the expressions of IGF-1R, SCF, and C-Kit in the gastric tissues of the DGP rats were greatly reduced, and the number of ICCs also declined significantly. After the treatment with ACVO, the expressions of IGF-1, SCF and ICCs were significantly increased. In addition, the level of serum IGF-1 also increased with the increase of the dose, suggesting that ACVO could up-regulate the expressions of IGF-1/IGF-1R signaling and SCF/C-Kit to restore ICCs and further promote gastric movement.

CHAT, an excitatory neuron (32, 33), maintains gastric movement together with inhibitory neurons, and IGF-1 plays an important role in the development of neurons. In this study, the expression of IGF-1/IGF-1R in DGP rats was significantly reduced, and the normal survival of CHAT could not be maintained, which was a factor in the delay of gastric emptying in rats. After the treatment of ACVO, CHAT expression was restored and increased with the increase of the dose of ACVO. The up-regulation of CHAT might be related to IGF-1/IGF-1R signaling which needs to be further explored.

## Conclusion

In general, ACVO increased body weight and decreased blood glucose in the DGP rats. ACVO effectively promoted gastric emptying and intestinal propulsion in the DGP rats and increased gastric acid secretion. Furthermore, after the treatment of ACVO, the serum IFG-1 level in DGP rats was increased, and the protein expressions and positive cell expressions of IGF-1, CHAT, and SCF in the gastric tissue were significantly increased. The morphology and arrangement of the cells and the number of ICCs in the gastric tissue were restored. ACVO plays a positive role in the treatment of DGP, and its mechanism may be related to increment of serum IGF-1 and up-regulation of IGF-1R in the gastric tissues, thereby up-regulating the expression of SCF, further restoring the number of ICCs, and promoting gastric movement. Meanwhile, the gastric expression of CHAT was restored and gastric movement was increased. The above research provides further insight into the potential role of ACVO in the development of DGP.

## Authors’ Contributions

HL, YW, FT, and ZX Conceived the study and design; YW Analyzed the data and prepared the draft manuscript; YG and MY Critically revised the paper; YG Supervised the research; HL and YW Approved the final version to be published.

## Conflicts of Interest

None of the authors has personal or financial conflicts of interest.
